# Enhancing the Prediction of Inborn Errors of Immunity: Integrating Jeffrey Modell Foundation Criteria with Clinical Variables Using Machine Learning

**DOI:** 10.3390/children12091259

**Published:** 2025-09-19

**Authors:** Alaaddin Yorulmaz, Ali Şahin, Gamze Sonmez, Fadime Ceyda Eldeniz, Yahya Gül, Mehmet Ali Karaselek, Şükrü Nail Güler, Sevgi Keleş, İsmail Reisli

**Affiliations:** 1 Department of Pediatrics, Selçuk University Medical School, Konya 42250, Turkey; 2 Department of Emergency Service, Dr. Vefa Tanır Ilgın State Hospital, Konya 42600, Turkey; sahin@silicosome.com; 3 Hacettepe University School of Medicine, Ankara 06100, Turkey; sonmez.gamze@hacettepe.edu.tr; 4 Department of Pediatric Immunology and Allergy, Medicine Faculty, Necmettin Erbakan University, Konya 42140, Turkey; f.eldeniz@saglik.gov.tr (F.C.E.); yahya.palu@hotmail.com (Y.G.); snguner@erbakan.edu.tr (Ş.N.G.); skeles@erbakan.edu.tr (S.K.); ireisli@erbakan.edu.tr (İ.R.)

**Keywords:** inborn errors of immunity, Jeffrey Modell Foundation, machine learning, support vector machine, clinical decision support systems

## Abstract

**Background**: Inborn errors of immunity (IEIs) are a heterogeneous group of rare disorders caused by genetic defects in one or more components of the immune system. The Jeffrey Modell Foundation’s (JMF) Ten Warning Signs are widely used for early detection; however, their diagnostic sensitivity is limited. Machine learning (ML) approaches may improve prediction accuracy by integrating additional clinical variables into decision-making frameworks. **Methods**: This retrospective study included 298 participants (98 IEI, 200 non-IEI) evaluated at a university-affiliated clinical immunology clinic between January and December 2020. IEI diagnoses were confirmed using European Society for Immunodeficiencies (ESID) criteria. Two datasets were constructed: one containing only JMF criteria and another combining JMF criteria with additional clinical variables. Four ML algorithms—random forest (RF), k-nearest neighbors (k-NN), support vector machine (SVM), and naive Bayes (NB)—were trained and optimized using nested 5-fold stratified cross-validation repeated three times. Performance metrics included accuracy, sensitivity, specificity, F1 score, Youden Index, and the area under the receiver operating characteristic curve (AUROC). SHapley Additive exPlanations (SHAP) were applied to evaluate feature importance. **Results**: Using only JMF criteria, the best-performing model was SVM (accuracy: 0.90 ± 0.04, sensitivity: 0.93 ± 0.05, AUROC: 0.91 ± 0.02). With the addition of clinical variables, the SVM achieved superior performance (accuracy: 0.94 ± 0.03, sensitivity: 0.97 ± 0.03, AUROC: 0.99 ± 0.00), outperforming both the classical JMF criteria (accuracy: 0.91, sensitivity: 0.87, AUROC: 0.90) and the JMF-only SVM model. SHAP analysis identified family history of early death, pneumonia history, and ICU admission as the most influential predictors. **Conclusions**: ML models, particularly SVM integrating JMF criteria with additional clinical variables, substantially improve IEI prediction compared with classical JMF criteria. Implementation of such models in clinical settings may facilitate earlier diagnosis and timely intervention, potentially reducing morbidity and healthcare burden in IEI patients.

## 1. Introduction

Inborn errors of immunity (IEIs) constitute a heterogeneous group of disorders caused by functional impairments in one or more components of the immune system, leading to insufficient or dysregulated immune responses [1]. To date, pathogenic variants in 508 genes and 17 phenocopies have been associated with IEIs [2]. Advances in modern diagnostic tools and genomic technologies continue to expand the list of genes implicated in these conditions [3]. Despite this growing genetic landscape, IEIs are still classified as rare diseases. Globally, the estimated prevalence of IEIs is approximately 1 in 1200 individuals, whereas in some Asian countries, the prevalence has been reported as 3.9 per 100,000 [4,5]. Patients with IEIs are particularly vulnerable. In addition, these conditions impose a substantial economic burden, underscoring their relevance as a public health concern.

One of the major challenges in the clinical management of IEIs is the delay in diagnosis, often due to limited awareness among healthcare professionals. Such delays can lead to suboptimal treatment, increased morbidity, and higher mortality rates [6]. Therefore, early recognition of IEIs is crucial for improving patient outcomes. To address this, several clinical warning systems have been developed to assist in the early identification of individuals with suspected immune deficiencies.

Among the most widely used tools for this purpose are the Jeffrey Modell Foundation’s (JMF) Ten Warning Signs, the German Patients’ Organization for Primary Immunodeficiencies (DSAI) criteria, and the Association of the Scientific Medical Societies in Germany (AWMF) and Düsseldorf Warning Signs [7,8,9,10]. These tools aim to provide simple yet effective clinical criteria for predicting the likelihood of an underlying IEI. Their primary objective is to facilitate the identification of affected individuals within large populations, thereby enabling timely referral and diagnostic work-up. Of these, the JMF warning signs have become one of the most commonly adopted and internationally recognized clinical tools in the early detection of IEIs [7].

Although the JMF warning signs are among the most commonly used clinical criteria for the early identification of IEIs, they may be insufficient in detecting certain cases [11]. In this study, we aimed to evaluate the diagnostic performance of classical JMF criteria within our cohort and to explore whether machine learning (ML) models based on these criteria could enhance predictive accuracy. Furthermore, we sought to investigate whether the integration of additional clinical features into the models could further improve the performance of these algorithms in predicting the presence of IEIs.

## 2. Methods

### 2.1. Ethical Considerations

Ethical approval for this study was obtained from the Ethics Committee of Necmettin Erbakan University, School of Medicine (Decision No: 2024/4828). Written informed consent was obtained from the legal guardians of all participants prior to inclusion in the study.

### 2.2. Study Design and Population

Between January 2020 and December 2020, individuals who presented to a university-affiliated clinical immunology outpatient clinic were retrospectively evaluated. Based on a comprehensive review of their clinical records, patients were classified into two groups: IEI and non-IEI. The eligibility of individuals in the IEI group was determined according to the diagnostic criteria established by the European Society for Immunodeficiencies (ESID). Patients who did not fulfill the ESID diagnostic criteria or had incomplete clinical data were excluded from the analysis. There were no missing values for any predictors or outcomes used in the analyses. Additionally, individuals with HIV infection or secondary immunodeficiencies (e.g., due to immunosuppressive therapy, malignancy, or chronic diseases) were also excluded to avoid potential confounding.

Demographic variables (including age and sex), the presence or absence of JMF warning signs, and other relevant clinical features (as detailed in Table 1) were extracted from the institutional electronic health records and compiled into a structured dataset for further analysis.

### 2.3. Machine Learning Model Development

In this study, we aimed to predict the presence of IEI by utilizing both JMF criteria and JMF criteria plus clinical data through the application of supervised ML algorithms. All analyses were performed using Python version 3.11.5 along with standard scientific libraries including pandas (v2.1.1) for data manipulation, numpy (v1.26.0) for numerical operations, scikit-learn (v1.3.1) for model development and evaluation, and matplotlib (v3.8.0) for data visualization.

To ensure an unbiased assessment of model performance, patients were randomly allocated into training (80%; n = 239) and test (20%; n = 59) sets. Stratified sampling was employed to preserve the class distribution of IEI and non-IEI cases within both subsets. This partitioning procedure was repeated independently for each algorithm to maintain consistency. Furthermore, stratification was preserved throughout all cross-validation folds to avoid potential bias arising from class imbalance.

We evaluated four commonly used supervised classification algorithms: random forest (RF), k-nearest neighbors (k-NN), support vector machine (SVM), and naive Bayes (NB). For each classifier, hyperparameter optimization was performed using RandomizedSearchCV. The parameter ranges were defined through ParameterGrid to ensure comprehensive search coverage while enabling computational efficiency through random sampling of the space. The hyperparameters explored included:

- for k-NN, the number of neighbors (3, 5, 7, 9) and weighting scheme (‘uniform’, ‘distance’);
- for SVM, the regularization parameter C (0.1, 1, 10), kernel type (‘linear’, ‘rbf’), and kernel coefficient gamma (‘scale’, ‘auto’);
- for RF, the number of estimators (100, 200, 500), maximum depth (None, 10, 20), and the number of features considered at each split (‘sqrt’, ‘log2’);
- for NB, the smoothing parameter var_smoothing (1 × 10^−9^, 1 × 10^−8^, 1 × 10^−7^).

Hyperparameter tuning was carried out using nested cross-validation with a 5-fold stratified approach repeated three times (i.e., 15 folds in total), implemented via RepeatedStratifiedKFold. The primary optimization metric was the area under the receiver operating characteristic curve (AUROC), selected for its robustness in evaluating binary classifiers, especially in datasets with class imbalance. For each algorithm, the hyperparameter configuration yielding the highest mean AUROC across the validation folds was selected for final model training.

Following optimization, each model was retrained on the full training dataset using the best-performing hyperparameters and subsequently evaluated on the independent test set. To ensure the robustness and generalizability of performance estimates, the entire training, validation, and testing pipeline was repeated 15 times for each classifier. This iterative approach allowed for the generation of a distribution of performance metrics, providing a more reliable basis for comparing model accuracy.

Finally, we examined feature contributions across all models and repetitions to identify variables that consistently demonstrated high predictive importance. Clinical variables and JMF-based features that emerged as key predictors may have future utility in informing diagnostic strategies and enhancing the performance of clinical decision support systems designed to facilitate the early detection of IEI.

### 2.4. Model Evaluation and Performance Metrics

To evaluate the predictive performance of the developed ML models, several standard classification metrics were calculated, including accuracy, sensitivity (recall), specificity, F1 score, and the AUROC. These metrics were computed for each model and for each grade category, across all cross-validation folds and repetitions. Receiver operating characteristic (ROC) curves were also generated to visually assess the diagnostic performance of each model.

For each model, the mean and standard deviation of the performance metrics were calculated across all iterations, enabling robust comparison between classifiers. Additionally, to gain insight into model interpretability and identify the most influential features in predicting IEI status, we applied SHapley Additive exPlanations (SHAP). Feature importance scores derived from SHAP values and model-intrinsic weight analyses were used to highlight the clinical parameters contributing most significantly to model predictions.

### 2.5. Statistical Analysis

Descriptive statistics were used to summarize the study cohort. Categorical variables were reported as frequencies and percentages, and group comparisons were performed using the chi-square test. Continuous variables were expressed as mean ± standard deviation (SD) along with 95% confidence intervals (CIs). The Kolmogorov–Smirnov test was used to assess the normality of data distribution. For comparisons between two groups, Student’s *t*-test was applied to normally distributed variables, while the Mann–Whitney U test was used for non-normally distributed variables. A two-tailed *p*-value < 0.05 was considered statistically significant. All statistical analyses were conducted using DATAtab software, https://numiqo.com/ (DATAtab GmbH, Graz, Austria).

## 3. Results

### 3.1. Patient Characteristics

298 participants were included in the study, consisting of 98 IEIs and 200 controls. The average age of the participants was 74.93 ± 62.59 months, with 146 females and 152 males. There was no significant sex differences between IEI and non-IEI groups. IEI participants were classified and included according to the International Union of Immunodeficiency Society (IUIS) 2024 system. IEI participants were most commonly comprising antibody deficiencies (Table S1).

### 3.2. Clinical and JMF Features of Participants

The JMF criteria and clinical features of the participants were evaluated and presented in Table 1 and Table 2. There was a significant association and difference between IEI and non-IEI groups in terms of total JMF scores (3.37 ± 1.66 vs. 0.34 ± 0.61; *p* < 0.001) (Table 1). IEI participants met the criteria for each JMF criterion significantly more than non-IEI participants (Table 1). In the JMF criteria, the criterion with the most significant difference between IEI and non-IEI participants was the need for intravenous (IV) antibiotics (82.65% vs. 15%; *p* < 0.001). In the IEI group, the percentage of participants not meeting at least 2 criteria from the JMF criteria was 12.24%. Additionally, in the non-IEI group, the percentage of participants meeting at least 2 criteria was 6.5% (Table 2).

The presence, number, and duration of hospitalizations were higher in the IEI group compared to the non-IEI group, and there was a significant difference. Furthermore, the frequency of being in the intensive care unit was more common in the IEI group compared to the non-IEI group (43.88% vs. 3%; *p* < 0.001). In addition to the otitis, pneumonia, and sinusitis parameters in the JMF criteria, the average number of these parameters per year was also evaluated, and it was observed that the average annual occurrence of these parameters was higher in the IEI group compared to the non-IEI group. (Table 1) Additionally, the average number of herpes labialis episodes per year was significantly higher in the IEI group compared to the non-IEI group (1.12 ± 2.49 vs. 0.03 ± 0.19; *p* < 0.001). The rate of post-vaccination complications was significantly higher in the IEI group compared to the non-IEI group, and the rate of lymphadenopathy following BCG vaccination was also higher in the IEI group compared to the non-IEI group. (Table 1) However, in our cohort, there was no significant difference between the groups in terms of the occurrence of discharge following BCG vaccination. (Table 1) The frequency of chronic skin problems among participants was significantly higher in the IEI group compared to the non-IEI group. (Table 1) The day of umbilical cord detachment was significantly higher in the non-IEI group compared to the IEI group (8.56 ± 4.52 vs. 7.1 ± 2.32; *p* < 0.001). The counterintuitive distribution of delayed cord separation warrants caution in interpretation. Because leukocyte adhesion deficiency is uncommon and cord-care/delivery factors can delay separation in healthy infants, this feature may be weakly specific for IEI in unselected populations and is best considered alongside other clinical indicators. In the IEI group, there was a significantly higher rate of delayed shedding of deciduous teeth and delayed wound healing compared to the non-IEI group. (Table 1) The occurrence rate of seizures, congenital heart disease, and chronic diarrhea was more frequent in IEI participants compared to the non-IEI group (Table 1).

The rate of consanguinity among parents was more frequent in the IEI group compared to the non-IEI group, and additionally, the average degree of consanguinity was significantly higher in the IEI group compared to the non-IEI group. (Table 1) The incidence of a family history of early death, tuberculosis activation, autoimmunity, and cancer were higher in the IEI group, while the incidence of allergies in the family was more frequent in the non-IEI group (Table 1).

### 3.3. The Performance of Machine Learning Models

ML algorithms are commonly used for classification and regression problems, and they often demonstrate superiority over basic prediction tools. In our study, we developed 8 different ML models using four different ML algorithms on two datasets created from the same participants (JMF criteria and additional clinical data beyond JMF criteria). To compare the effectiveness of the developed ML models in distinguishing between IEI and non-IEI, we calculated six different performance metrics. Performance metrics were computed for each repeat and cross-validation of the ML models, and the mean and standard deviation of the performance metrics were calculated.

#### 3.3.1. Accuracy

One of the most widely used metrics for assessing the performance of ML models is accuracy, which is defined as the proportion of correctly classified instances to the total number of predictions made. In predicting the presence of IEI, the classical JMF criteria alone achieved an accuracy of 0.91. Among the ML models developed using only the JMF criteria, the k-NN algorithm achieved the highest accuracy (0.91 ± 0.04). When additional clinical variables were incorporated alongside the JMF criteria, the highest accuracy was obtained with the SVM model (0.94 ± 0.03) (Table 3).

#### 3.3.2. F1 Score

The F1 score is another key performance metric for evaluating ML models, defined as the harmonic mean of precision and recall, and particularly useful in scenarios involving class imbalance. Using the classical JMF warning signs alone, the F1 score was 0.87. Among the ML models developed solely on the basis of JMF criteria, the highest F1 scores were achieved by the k-NN model (0.86 ± 0.06) and the SVM model (0.86 ± 0.05). When additional clinical variables were incorporated alongside the JMF criteria, the SVM model achieved the highest F1 score (0.92 ± 0.04) (Table 3).

#### 3.3.3. Sensitivity

When evaluating the sensitivity of the classical JMF criteria, the JMF-based ML models, and the ML models developed by integrating additional clinical variables with the JMF criteria, the classical JMF criteria demonstrated a sensitivity of 0.87. Among the JMF-based ML models, the SVM achieved the highest sensitivity (0.93 ± 0.05). In the models incorporating additional clinical variables alongside the JMF criteria, the SVM again exhibited the best performance, with a sensitivity of 0.97 ± 0.03 (Table 3).

#### 3.3.4. Specificity

When evaluating specificity metrics, the classical JMF criteria achieved a specificity of 0.93. Among the ML models developed using only the JMF criteria, the NB model demonstrated the highest specificity (0.95 ± 0.02). In contrast, when additional clinical variables were incorporated alongside the JMF criteria, the highest specificity was achieved by the k-NN model (0.99 ± 0.01) (Table 3).

#### 3.3.5. Youden Index

To cumulatively assess sensitivity and specificity, we also calculated the Youden Index (YI), defined as (sensitivity + specificity − 1). The classical JMF criteria yielded a YI of 0.81. Among the ML models developed solely on the basis of JMF criteria, the highest YI was achieved by the SVM model (0.81 ± 0.07). When additional clinical variables were incorporated into the JMF-based models, the SVM again demonstrated the highest YI, reaching 0.90 ± 0.04 (Table 3).

#### 3.3.6. AUROC

**Figure 1:**
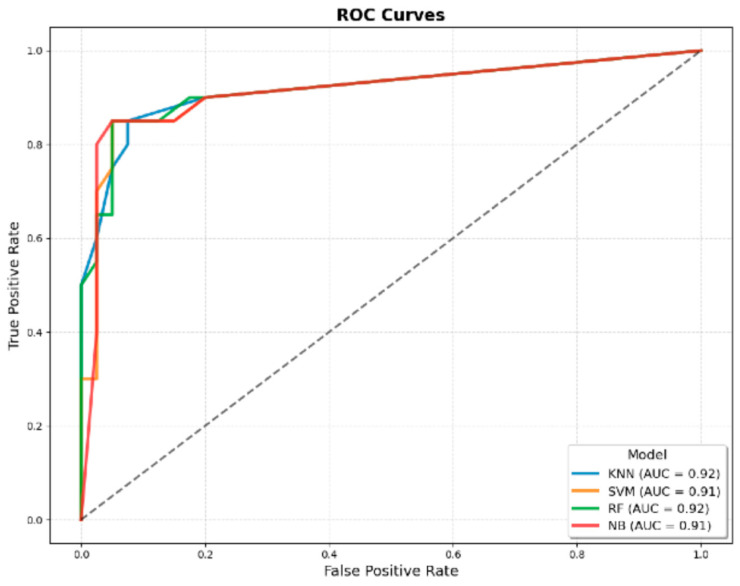
ROC curves of machine learning models developed using only JMF criteria for predicting IEI. The four evaluated algorithms were KNN, SVM, RF, and NB. The AUROC values were 0.92 for KNN, 0.91 for SVM, 0.92 for RF, and 0.91 for NB, indicating comparable discriminatory performance across models.

**Figure 2:**
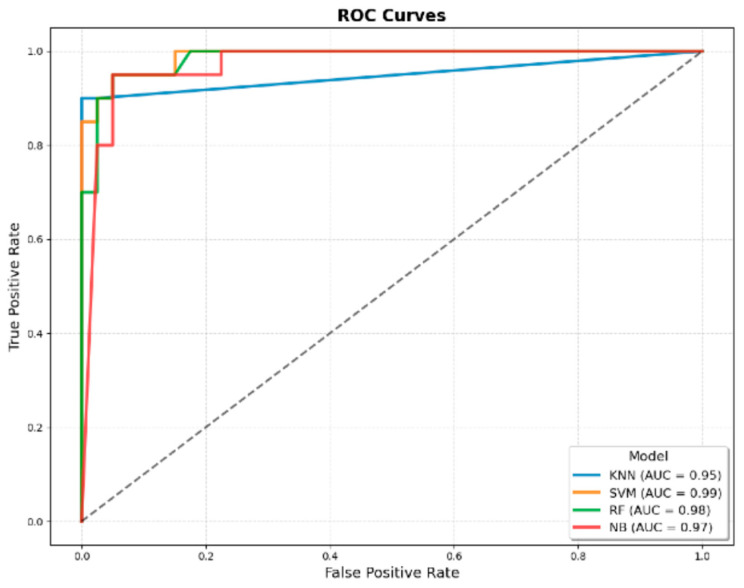
ROC curves of machine learning models developed using JMF criteria combined with additional clinical variables for predicting IEI. The four evaluated algorithms were KNN, SVM, RF, and NB. The AUROC values were 0.95 for KNN, 0.99 for SVM, 0.98 for RF, and 0.97 for NB, demonstrating superior discriminatory performance compared with models developed using only JMF criteria.

When comparing the AUROC values of the classical JMF criteria, the JMF-based ML models, and the models developed by integrating additional clinical variables with the JMF criteria, the AUROC for the classical JMF criteria was 0.90. Among the ML models developed using only the JMF criteria, the highest AUROC values were achieved by the k-NN model (0.92 ± 0.03) and the RF model (0.92 ± 0.03) (Figure 1 and Table 3). In contrast, when additional clinical variables were incorporated into the JMF-based dataset, the SVM model achieved the highest AUROC (0.99 ± 0.00) (Figure 2 and Table 3).

#### 3.3.7. Comparison of the Classical JMF Criteria with the Best-Performing JMF-Based ML Model

When comparing the classical JMF criteria with ML models developed using only JMF features, the SVM demonstrated the best performance among the JMF-based models (AUROC: 0.91 ± 0.02, Accuracy: 0.90 ± 0.04, Sensitivity: 0.93 ± 0.05, Specificity: 0.89 ± 0.05, F1 score: 0.86 ± 0.05, YI: 0.81 ± 0.07). Relative to the classical JMF criteria (AUROC: 0.90, Accuracy: 0.91, Sensitivity: 0.87, Specificity: 0.93, F1 score: 0.87, YI: 0.81), the SVM model achieved a notable improvement in sensitivity while maintaining comparable accuracy and F1 score, albeit with a slight reduction in specificity (Table 3).

#### 3.3.8. Comparison of the Best JMF-Based ML Model with the Best Integrated Model (JMF + Additional Clinical Variables)

When additional clinical variables were incorporated alongside the JMF criteria, the SVM again emerged as the best-performing model (AUROC: 0.99 ± 0.00, Accuracy: 0.94 ± 0.03, Sensitivity: 0.97 ± 0.03, Specificity: 0.93 ± 0.05, F1 score: 0.92 ± 0.04, YI: 0.90 ± 0.04). Compared with the SVM trained solely on JMF criteria, the integrated model achieved consistent improvements across all performance metrics, including marked increases in AUROC (+0.08), accuracy (+0.04), sensitivity (+0.04), and YI (+0.09), while maintaining specificity at a similar level.

#### 3.3.9. Comparison of the Classical JMF Criteria with the Best Integrated Model (JMF + Additional Clinical Variables)

Direct comparison between the classical JMF criteria and the integrated SVM model demonstrated clear superiority of the latter across all evaluated metrics. AUROC increased from 0.90 to 0.99, accuracy from 0.91 to 0.94, sensitivity from 0.87 to 0.97, and F1 score from 0.87 to 0.92, with the YI rising from 0.81 to 0.90, while specificity remained constant at 0.93. These findings indicate that incorporating additional clinical variables into JMF-based ML models substantially enhances diagnostic performance in predicting the presence of IEI.

### 3.4. Feature Importance Analysis of the Integrated SVM Model Using SHAP Values

**Figure 3:**
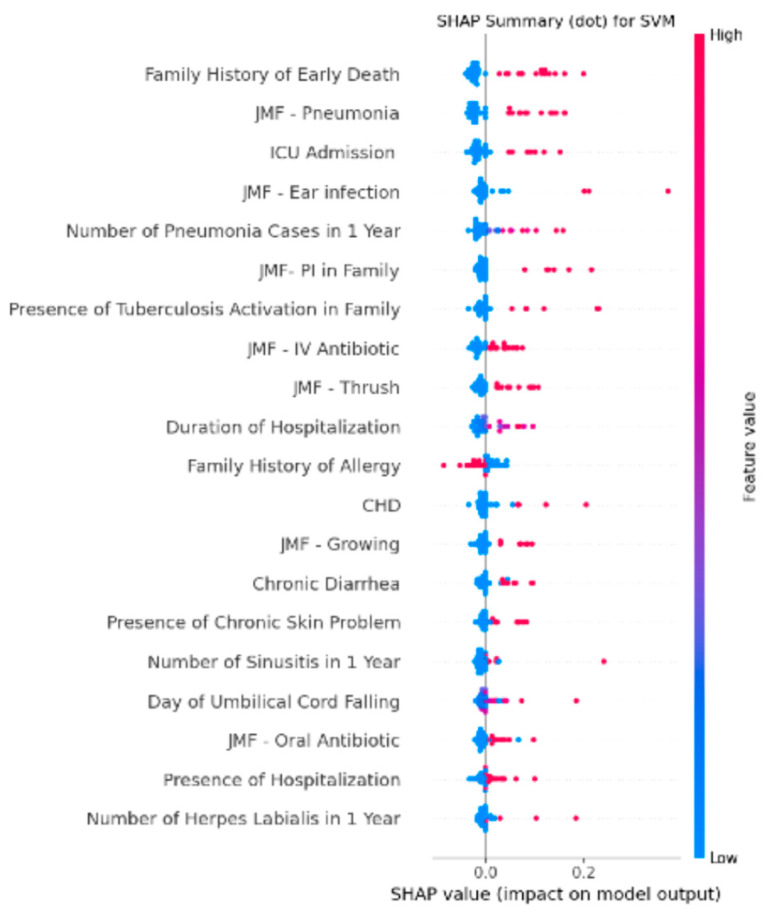
SHAP summary plot of the best-performing SVM model developed using JMF criteria combined with additional clinical variables for predicting IEI. The plot ranks features by their average absolute SHAP value, indicating their relative contribution to model predictions. The most influential predictors were family history of early death, JMF–pneumonia criterion, ICU admission, JMF–ear infection criterion, and number of pneumonia cases within one year. Color denotes the original feature value (red = high, blue = low), and position along the x-axis reflects the impact on the model output.

SHAP analysis of the best-performing model (SVM trained with JMF criteria and additional clinical variables) revealed that the most influential predictors for IEI classification were family history of early death, history of pneumonia according to JMF criteria, and ICU admission. Other variables with substantial contributions included JMF—ear infection, number of pneumonia episodes within one year, primary immunodeficiency in the family, and presence of tuberculosis activation in the family. Clinical parameters such as IV antibiotic requirement (JMF), thrush (JMF), and duration of hospitalization also ranked among the top contributors. High SHAP values for these features were associated with an increased likelihood of IEI prediction, whereas features with lower SHAP values, such as number of herpes labialis episodes in one year and presence of hospitalization, had comparatively less impact on model output. These findings suggest that, in addition to classical JMF warning signs, certain clinical indicators reflecting severe or recurrent infections, family history, and hospitalization burden substantially enhance the discriminatory capacity of ML-based IEI prediction models (Figure 3).

## 4. Discussion

Inborn errors of immunity are disorders characterized by dysfunction or overactivation of the immune system of genetic origin [11,12]. As per the IUIS 2024 update, there are 508 genetic variants related to IEI identified, and new immunodeficiencies are being identified as days go by [2]. Various systems have been developed for both pediatric and adult patients to facilitate early detection of IEI and raise awareness [13,14]. These classification systems contribute to the early identification of IEI patients, enabling them to receive timely diagnosis and consequently benefit from more targeted and effective treatment strategies. This, in turn, enhances the overall prognosis and treatment outcomes for individuals afflicted with IEI. Additionally, early diagnosis of IEI patients can help reduce the financial burden on the healthcare system associated with managing IEI-related complications [6]. The most commonly used system for identifying the presence of IEI is the “10 Warning Signs of IEI” created by the non-profit organization, the JMF [7]. However, it is acknowledged that this system may be insufficient in recognizing the presence of IEI in some patients [14]. In a study conducted by Eldeniz et al., the JMF system failed to identify 12.2% of IEI patients [7]. The sensitivity of the JMF system in identifying IEI was 56%, while its specificity was 15% [14]. In our study, we aimed to utilize ML algorithms, which continue to be an indispensable part of modern medicine, to determine the presence of IEI.

ML algorithms are commonly used for regression, classification, and clustering problems, often yielding successful results. They serve as pioneers in systems frequently employed in modern medicine, assisting healthcare professionals. ML algorithms are extensively utilized in tasks such as disease diagnosis through the processing of radiological images, predicting post-operative complications, and cancer staging [15,16,17]. In the field of allergies, ML algorithms have been utilized for determining the effectiveness of subcutaneous immunotherapy, assessing the risk of anaphylaxis, and identifying the presence of pediatric asthma [18,19,20]. In the field of clinical immunology, ML techniques are less frequently used compared to the allergy domain. This may be attributed to the lower prevalence of allergic diseases compared to diseases relevant to clinical immunology. Generating a sufficiently large dataset can be more challenging in clinical immunology than in allergy. However, there are studies indicating the potential use of ML and AI models in clinical immunology for differential diagnosis of IEIs, ensuring early diagnosis of IEIs, and IEI screening [21,22,23].

In our study, we created two separate datasets using the JMF criteria and clinical data of 298 participants. The first dataset was constructed solely from the participants’ JMF criteria, while the second dataset included both the JMF criteria and additional clinical data. We developed a total of 8 ML models using four different algorithms simultaneously from both datasets (four models using only the JMF criteria and four models using the JMF criteria plus additional clinical data). We calculated the performance metrics of accuracy, sensitivity, specificity, F1 score, YI and AUROC for each ML model in every repeat and cross-validation. To determine which dataset and algorithm performed best in detecting IEI presence, we compared the performance metrics of the models with each other. Our model with the highest performance was the SVM model using the JMF criteria plus additional clinical data dataset. The SVM model had an accuracy of 0.94 ± 0.03, sensitivity of 0.97 ± 0.03, and specificity of 0.93 ± 0.05. The most effective features used by the SVM model developed with the JMF criteria plus additional clinical data for IEI detection were family history of early death, JMF-pneumonia criteria, ICU admission, JMF-ear infection criteria, and number of pneumonia cases in 1 year. Additionally, these features could be evaluated as triggering indicators for determining IEI presence more effectively.

In studies related to the determination of IEI presence, Mayampurath et al. developed ML models for the early diagnosis of IEI patients. In their study, when they incorporated laboratory data in addition to clinical data, they achieved an AUROC value of 0.72 [23]. Similarly to our study, they also reached the highest AUROC value using the SVM model (0.99 ± 0.00) × 20 Barrera, Jose Alfredo Méndez et al. developed ML models to differentiate IEI cases according to subtypes defined by the IUIS using the USIDNET database and succeeded in creating highly successful models. In their developed model, they achieved the highest accuracy (0.996) in detecting leukocyte adhesion defects, while they reached the highest AUROC value (0.89) in detecting DiGeorge syndrome [22]. Rider et al. developed an artificial intelligence-based IEI screening system [21]. In their developed system, they first identify high-risk cases for IEI using only the JMF criteria and then utilize AI to detect IEI patients. They achieved high performance metrics with their developed AI system.18 The most successful model had an accuracy of 1.0, an AUROC of 1.0, and an F1 score of 1.0.18 However, relying solely on the initial screening with JMF criteria in their developed system may lead to missing some IEI cases. This is because literature reports that JMF may incorrectly predict some IEI cases.

This study has several limitations. First, its retrospective, single-center design limits the generalizability of the findings to broader populations with differing demographic and clinical characteristics. Second, although the cohort is relatively large for an IEI-focused investigation, the overall sample, and several IEI subtype strata, remains modest, which may reduce the robustness of subgroup analyses and affect model stability and calibration. Third, we did not perform external validation; therefore, model performance in other healthcare settings remains unknown. Although we used multiple performance metrics and SHAP analysis to support interpretability (and employed rigorous internal validation as detailed above), such procedures cannot substitute for testing on truly independent cohorts. Fourth, laboratory and genetic data were not included in model development; incorporating these features may enhance predictive accuracy and transportability. Finally, we did not evaluate cost-effectiveness or real-world clinical impact, which warrants prospective assessment.

In particular, our predictors were not restricted to information available at the first clinical encounter, so some features may be unavailable at the time of decision-making. We envision this model as a provider-facing triage aid embedded in the electronic health record and used by front-line clinicians—e.g., primary care, pediatrics, pulmonology, dermatology, urgent care, and emergency medicine, where undiagnosed IEI often presents. The tool is intended to complement, not replace, the JMF warning signs and clinician judgment. In practice, it could be deployed (i) to prioritize referrals among patients who already meet JMF warning signs and (ii) to flag additional high-risk patients who do not meet all JMF criteria but exhibit suggestive patterns in routinely available data at the first encounter. It is not designed for unsupervised use by patients for self-referral. Future work will benchmark a minimal “first-encounter” feature set, co-develop clinically acceptable operating thresholds such as using decision-curve/net-benefit analysis under different referral capacities, and prospectively assess alert burden, referral yield, time to diagnosis, and downstream costs to determine whether model-assisted triage improves care.

In conclusion, we developed ML models that accurately identify patients with suspected IEI. However, given the retrospective, single-center design and the absence of external validation, these findings should be considered preliminary. The immediate next step is external validation in independent, multi-center prospective cohorts to assess discrimination, calibration, transportability, and potential need for model updating before any clinical deployment. Future work should also explore integrating laboratory and genetic data and formally evaluate clinical impact and cost-effectiveness to enhance applicability in routine practice.

## Figures and Tables

**Table 1:** The characteristics of clinical and JMF 10 warning sign criteria for IEI and non-IEI participants. (Abbreviations: IEI, inborn errors of immunity; n, number; ns, non significant; CI, confidence interval; BCG, Bacillus Calmette–Guérin; CHD, Congenital heart disease; ICU, intensive care unit; JMF, Jeffery Modell Foundation; α chi-square test applied; β Mann–Whitney U-test applied).

Features		Overall	IEI	Non-IEI	*p* Value
Patients, (n, %)		298 (100)	98 (32.8)	200 (67.2)	-
Age (month), (mean ± SD)		74.93 ± 62.59 [68.43–81.44]	98.87 ± 66.37 [85.56–112.17]	65.46 ± 63.71 [56.53–74.38]	**<0.001 ^α^**
Sex	Female, (n, %)	146 (48.99)	48 (48.98)	98 (49)	ns ^α^
	Male, (n, %)	152 (51.01)	50 (51.02)	102 (51)	
**JMF Warning Signs**					
≥4 new ear infections within 1 year, (n, %)		9 (3.02)	8 (8.16)	1 (0.5)	**<0.001 ^α^**
≥2 serious sinus infections within 1 year, (n, %)		11 (3.69)	9 (9.18)	2 (1)	**<0.001 ^α^**
≥2 months on antibiotics with little effect, (n, %)		53 (17.79)	45 (45.92)	8 (4)	**<0.001 ^α^**
≥2 pneumonias within 1 year, (n, %)		70 (23.49)	63 (64.29)	7 (3.5)	**<0.001 ^α^**
Failure of an infant to gain weight or grow normally, (n, %)		40 (13.42)	37 (37.76)	3 (1.5)	**<0.001 ^α^**
Recurrent, deep skin or organ abscesses, (n, %)		10 (3.36)	9 (9.18)	1 (0.5)	**<0.001 ^α^**
Persistent thrush in mouth or fungal infection on skin, (n, %)		55 (18.46)	43 (43.88)	12 (6)	**<0.001 ^α^**
Need for IV antibiotics to clear infections, (n, %)		111 (37.25)	81 (82.65)	30 (15)	**<0.001 ^α^**
≥2 deep-seated infections including septicemia, (n, %)		10 (3.36)	9 (9.18)	1 (0.5)	**<0.001 ^α^**
Family history of IEI, (n, %)		29 (9.73)	25 (25.51)	4 (2)	**<0.001 ^α^**
Total JMF points, (mean ± SD) [95% CI]		1.34 ± 1.78 [1.13–1.54]	3.37 ± 1.66 [3.03–3.7]	0.34 ± 0.61 [0.25–0.43]	**<0.001 ^β^**
**Additional Clinical Data**					
Presence of Hospitalization, (n, %)		130 (43.62)	86 (87.76)	44 (12.24)	**<0.001 ^α^**
Number of Hospitalizations, (mean ± SD) [95% CI]		7.07 ± 18.68 [4.93–9.21]	12.83 ± 25.42 [7.73–17.92]	4.25 ± 13.47 [2.36–6.13]	**<0.001 ^β^**
Duration of Hospitalization, (mean ± SD) [95% CI]		4.87 ± 6.33 [4.15–5.6]	10.69 ± 7.43 [9.2–12.18]	2.02 ± 2.85 [1.62–2.42]	**<0.001 ^β^**
Number of Otitis in 1 Year, (mean ± SD) [95% CI]		0.42 ± 1.37 [0.26–0.57]	1.07 ± 2.16 [0.64–1.5]	0.1 ± 0.47 [0.03–0.16]	**<0.001 ^β^**
Number of Sinusitis in 1 Year, (mean ± SD) [95% CI]		0.24 ± 0.94 [0.13–0.35]	0.59 ± 1.38 [0.31–0.87]	0.07 ± 0.54 [-0.01–0.15]	**<0.001 ^β^**
Number of Pneumonia in 1 Year, (mean ± SD) [95% CI]		1.37 ± 2.58 [1.07–1.66]	3.65 ± 3.42 [2.97–4.34]	0.25 ± 0.6 [0.17–0.33]	**<0.001 ^β^**
Number of Herpes Labialis in 1 Year, (mean ± SD) [95% CI]		0.39 ± 1.52 [0.21–0.56]	1.12 ± 2.49 [0.62–1.62]	0.03 ± 0.19 [0–0.05]	**<0.001 ^β^**
Vaccination Related Complications, (n, %)		8 (2.68)	6 (6.12)	2 (1)	**<0.05 ^α^**
Discharge After BCG Vaccination, (n, %)		5 (1.68)	3 (3.06)	2 (1)	ns ^α^
Lymphadenopathy After BCG Vaccination, (n, %)		3 (1.01)	3 (1.01)	0 (0)	**<0.05 ^α^**
Presence of Chronic Skin Problem, (n, %)		55 (18.46)	44 (44.9)	11 (5.5)	**<0.001 ^α^**
Day of Umbilical Cord Falling, (mean ± SD) [95% CI]		7.58 ± 3.28 [7.2–7.95]	7.1 ± 2.32 [6.77–7.42]	8.56 ± 4.52 [7.66–9.47]	**<0.001 ^β^**
Delay in Milk Tooth Shedding, (n, %)		16 (5.37)	12 (12.24)	4 (2)	**<0.001 ^α^**
Delay in Wound Healing, (n, %)		35 (11.74)	29 (29.59)	6 (3)	**<0.001 ^α^**
Convulsion, (n, %)		26 (8.72)	20 (20.41)	6 (3)	**<0.001 ^α^**
CHD, (n, %)		23 (7.72)	21 (21.43)	2 (1)	**<0.001 ^α^**
Chronic Diarrhea, (n, %)		34 (11.41)	28 (28.57)	6 (3)	**<0.001 ^α^**
ICU Admission, (n, %)		49 (16.44)	43 (43.88)	6 (3)	**<0.001 ^α^**
Presence of Consanguinity Between Parents, (n, %)		21 (7.05)	16 (16.33)	5 (2.5)	**<0.001 ^α^**
Degree of Consanguinity, (mean ± SD) [95% CI]		0.28 ± 0.69 [0.2–0.36]	0.57 ± 0.86 [0.4–0.74]	0.14 ± 0.53 [0.06–0.21]	**<0.001 ^β^**
Family History of Early Death, (n, %)		77 (25.84)	66 (67.35)	11 (5.5)	**<0.001 ^α^**
Presence of Tuberculosis Activation in Family, (n, %)		21 (7.05)	16 (16.33)	5 (2.5)	**<0.001 ^α^**
Family History of CHD, (n, %)		25 (8.39)	11 (11.22)	14 (7)	ns ^α^
Family History of Autoimmunity, (n, %)		51 (17.11)	28 (28.57)	23 (11.5)	**<0.001 ^α^**
Family History of Allergy, (n, %)		129 (43.29)	31 (31.63)	98 (49)	**<0.05 ^α^**
Family History of Cancer, (n, %)		52 (17.45)	28 (28.57)	24 (12)	**<0.001 ^α^**

**Table 2:** The distribution of JMF scores for IEI and non-IEI groups. (Abbreviations: JMF, Jeffery Modell Foundation; IEI, Inborn Errors of Immunity; α chi-square test applied).

Total JMF Points	Overall	IEI	Non-IEI	*p* Value ^α^
0 points	148 (49.66)	2 (2.04)	146 (73)	**<0.001**
1 points	51 (17.11)	10 (10.2)	41 (20.5)	
2 points	34 (11.41)	22 (22.45)	12 (6)	
3 points	21 (7.05)	19 (19.39)	1 (0.5)	
4 points	20 (6.71)	21 (21.43)	0 (0)	
5 points	13 (4.36)	13 (13.27)	0 (0)	
6 points	7 (2.35)	7 (7.14)	0 (0)	
7 points	4 (1.34)	4 (4.08)	0 (0)	
8 points	0 (0)	0 (0)	0 (0)	
9 points	0 (0)	0 (0)	0 (0)	
10 points	0 (0)	0 (0)	0 (0)	

**Table 3:** Performance metrics of the classical JMF criteria, ML models developed using only JMF features, and ML models developed using JMF features combined with additional clinical variables for predicting IEI. The four evaluated ML algorithms were KNN, SVM, RF, and NB. Metrics include accuracy, sensitivity, specificity, F1 Score, Youden Index, and AUROC. Values are reported as mean ± SD across all cross-validation folds and repetitions for ML models, and as single values for the classical JMF criteria.

	Classical JMF Criteria	ML Models (Using Only JMF Features)				ML Models (Using JMF + Additional Features)			
Metrics	Threshold-Based Scoring	KNN	SVM	RF	NB	KNN	SVM	RF	NB
Accuracy	0.91	0.91 ± 0.04	0.90 ± 0.04	0.90 ± 0.03	0.88 ± 0.03	0.85 ± 0.03	0.94 ± 0.03	0.93 ± 0.03	0.93 ± 0.03
Sensitivity	0.87	0.83 ± 0.06	0.93 ± 0.05	0.84 ± 0.08	0.73 ± 0.10	0.56 ± 0.08	0.97 ± 0.03	0.84 ± 0.07	0.93 ± 0.04
Specificity	0.93	0.94 ± 0.04	0.89 ± 0.05	0.93 ± 0.04	0.95 ± 0.02	0.99 ± 0.01	0.93 ± 0.05	0.98 ± 0.02	0.94 ± 0.04
F1 Score	0.87	0.86 ± 0.06	0.86 ± 0.05	0.85 ± 0.05	0.80 ± 0.07	0.71 ± 0.07	0.92 ± 0.04	0.89 ± 0.05	0.90 ± 0.04
Youden Index	0.81	0.78 ± 0.08	0.81 ± 0.07	0.77 ± 0.08	0.68 ± 0.10	0.56 ± 0.08	0.90 ± 0.04	0.82 ± 0.08	0.86 ± 0.05
AUROC	0.90	0.92 ± 0.03	0.91 ± 0.02	0.92 ± 0.03	0.91 ± 0.02	0.95 ± 0.03	0.99 ± 0.00	0.98 ± 0.01	0.97 ± 0.02

## Data Availability

The data that support the findings of this study are available from the corresponding author upon reasonable request. The data are not publicly available due to privacy.

## Supplementary Materials

The following supporting information can be downloaded at: https://www.mdpi.com/article/10.3390/children12091259/s1, Table S1: The distribution of IEI group according to IEI subtypes based on IUIS classification. (2024) (Abbreviations: IUIS, International Union of Immunological Societies; n, number).
