# Supplementary material for: Enhancing the Prediction of Inborn Errors of Immunity: Integrating Jeffrey Modell Foundation Criteria with Clinical Variables Using Machine Learning
Source: Children (Basel). 2025 Sep 19;12(9):1259. doi: 10.3390/children12091259 (PMC12468333; doi:10.3390/children12091259)
Supplement: Supplementary file 1 [file children-12-01259-s001.zip › children-3842102-supplementary.pdf]

Table S1. The distribution of IEI group according to IEI subtypes based on IUIS classification. (2024) (Abbreviations: IUIS, International Union of Immunological Societies; n, number)

**IUIS Type**	**n**	**%**
Immunodeficiencies affecting cellular and humoral immunity	18	18.4
Combined immunodeficiencies with associated syndromic features	21	21.4
Immunodeficiencies due to antibody deficiencies	39	39.8
Diseases of immune dysregulation	7	7.1
Congenital defects of phagocyte number or function or both	8	8.2
Defects in Intrinsic and Innate Immunity	1	1
Auto-inflammatory disorders	3	3.1
Complement deficiencies	1	1
Bone marrow failure	0	0
Phenocopies of IEI	0	0
**Total**	98	100
